# Janus acoustic metascreen with nonreciprocal and reconfigurable phase modulations

**DOI:** 10.1038/s41467-021-27403-4

**Published:** 2021-12-06

**Authors:** Yifan Zhu, Liyun Cao, Aurélien Merkel, Shi-Wang Fan, Brice Vincent, Badreddine Assouar

**Affiliations:** Université de Lorraine, CNRS, Institut Jean Lamour, Nancy, 54000 France

**Keywords:** Acoustics, Metamaterials, Electrical and electronic engineering

## Abstract

Integrating different reliable functionalities in metastructures and metasurfaces has become of remarkable importance to create innovative multifunctional compact acoustic, optic or mechanical metadevices. In particular, implementing different wave manipulations in one unique material platform opens an appealing route for developing integrated metamaterials. Here, the concept of Janus acoustic metascreen is proposed and demonstrated, producing two-faced and independent wavefront manipulations for two opposite incidences. The feature of two-faced sound modulations requires nonreciprocal phase modulating elements. An acoustic resonant unit cell with rotating inner core, which produces a bias by a circulating fluid, is designed to achieve high nonreciprocity, leading to decoupled phase modulations for both forward and backward directions. In addition, the designed unit cell consisting of tunable phase modulators is reconfigurable. A series of Janus acoustic metascreens including optional combinations of extraordinary refraction, acoustic focusing, sound absorption, acoustic diffusion, and beam splitting are demonstrated through numerical simulations and experiments, showing their great potential for acoustic wavefront manipulation.

## Abstract (web summary)

Here, the authors introduce the concept of Janus acoustic metascreen for independent wavefront manipulations for two opposite incidences. They use acoustic circulators with rotating inner cores to achieve high nonreciprocity, and demonstrate tunable combinations of wavefront manipulations.

## Introduction

The god Janus in Roman mythology was seen as two-faced, looking to the past and the future, respectively. Inspired by this implication, scientists have named two-faced particles as Janus particle^1^, which possess different materials on the opposite sides. A similar concept called Janus monolayer has been applied in two-dimensional (2D) materials, which can obtain giant band gap tunability by breaking structural symmetry, leading to the development of electronic nanodevices^2,3^. Recently, the concept of Janus function has been extended to optical metasurfaces^4,5^. For example, the dynamic Janus metasurfaces^4^ can achieve dynamic and switchable optical functions, such as beam steering and holographic encryptions, whose tunability is realized by the chemical method. The directional Janus metasurfaces^5^ can realize two-faced and independent wave manipulations with two opposite incidences, such as asymmetrical wavefront manipulation and direction-controlled holograms. This extends the manipulation of conventional metasurfaces from one to two sides. So far, the acoustic counterpart of Janus metasurfaces has not been presented or demonstrated.

On the other hand, as a transformative concept in acoustics, acoustic metasurfaces^6^ with phase discontinuity at the interface between two mediums have the abilities to achieve various sound manipulations with subwavelength dimensions. Different acoustic wavefront manipulations have been demonstrated by acoustic metasurfaces or metascreens, such as extraordinary refraction^7^, acoustic focusing^8^, non-diffraction beam^9^, acoustic diffusion^10^, and so on. After that, various acoustic metasurfaces and metascreens with more complex physics or higher degree of freedoms have been demonstrated, such as bianisotropic unit cells^11,12^, asymmetric wavefront manipulations^13–15^, simultaneous amplitude and phase modulations^16,17^, and frequency multiplexed features^18,19^. However, another important wave property, *viz*., propagation direction, has rarely been exploited for multifunctional acoustic metasurfaces^20^. To date, most of the acoustic metasurfaces and metascreens can achieve identical functionalities only for one direction, leaving the directionality features unexploited.

In this research, we introduce and demonstrate the concept of Janus acoustic metascreen (JAM) with direction-encoded, two-faced wavefront manipulations. The key point for designing JAM lies on the requirement of high nonreciprocity within a compact unit cell, and the totally nonreciprocal phase modulation. Previous works on asymmetric wavefront manipulations^13–15^ with linear systems are essentially reciprocal for which the asymmetrical responses rely on wave-vector or diffraction modes conversion. However, freely and fully manipulation of two-faced acoustic wavefront is still a challenging problem. The number of strategies to break the reciprocity of acoustic wave propagation is limited. One can apply an external bias such as flow to the medium, utilize space–time modulated materials or consider nonlinear effects along with spatial asymmetry^21–33^. Considering the requirement of high efficiency and small volume for the unit cell of JAM, acoustic circulators^32,33^ can be used to break the time-reversal symmetries. Here, we theoretically and numerically show the realization of highly nonreciprocal transmission with the desired phase response, by introducing phase modulators in an acoustic resonator with rotating inner core. In addition, we propose experimental verifications with 3D-printed structures driven by motors to validate the design principle and its feasibility. For the convenience of experiment, the phase modulation part uses tunable element^34^ to realize reconfigurable phase modulated structures. We evidence JAMs that enable direction-controlled, two-faced wave manipulation with various combinations of two distinct functionalities, including extraordinary refraction, total transmission, total absorption, acoustic focusing, acoustic diffusion, and beam splitting. Such feature of encoding two functionalities in a single metasurface could lead to new acoustic conceptual devices, with the applications of nonreciprocal acoustic transmission and multifunctional sound manipulations.

## Results

### Nonreciprocal phase modulating unit cell

**Fig. 1:**
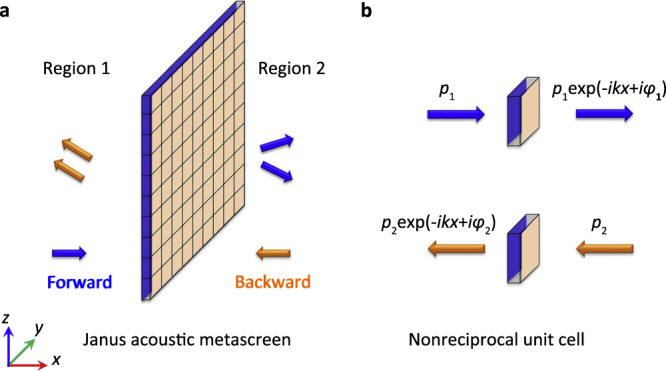
Concept of Janus acoustic metascreen. **a** Schematic diagram of the Janus acoustic metascreen. **b** Schematic diagram of the nonreciprocal phase modulating unit cell. The blue and orange arrows indicate the forward and backward directions.

As for classical metasurfaces, we are aiming at introducing a controlled additional phase shift to the transmitted wave. The unusual feature of a JAM resides on the fact that this phase shift depends on whether the incidence is in the forward direction (FD) or in the backward direction (BD) as illustrated by blue and orange arrows in Fig. 1a, respectively. Considering one single unit cell as shown in Fig. 1b and assuming the ideal case of total transmission, the incident plane wave in the forward (backward) direction has an acoustic pressure written as *p* = *p*_1_*e*^*−ikx*^ (*p* = *p*_2_*e*^*ikx*^), where *k* is the wave number *k* = *ω*/*c*, *c* is the sound speed *c* = 343 m/s and *ω* is angular frequency *ω* = 2*πf* with the frequency *f*. The transmitted acoustic pressure reads ${p_1}{e}^{{-ikx+i\varphi}_1}$ (${p_2}{e}^{{-ikx+i\varphi}_2}$) with a phase shift of *φ*_1_ (*φ*_2_), the metascreen being at the origin of the *x*-axis. In a reciprocal system, the relation *φ*_1_ = *φ*_2_ holds, as long as the unit cell remains subwavelength. On the contrary, we have the nonreciprocal relation *φ*_1_ ≠ *φ*_2_ in our design, where the respective values of *φ*_1_ and *φ*_2_ are independently controlled, which ensure the two-faced wavefront manipulations of JAM.

**Fig. 2:**
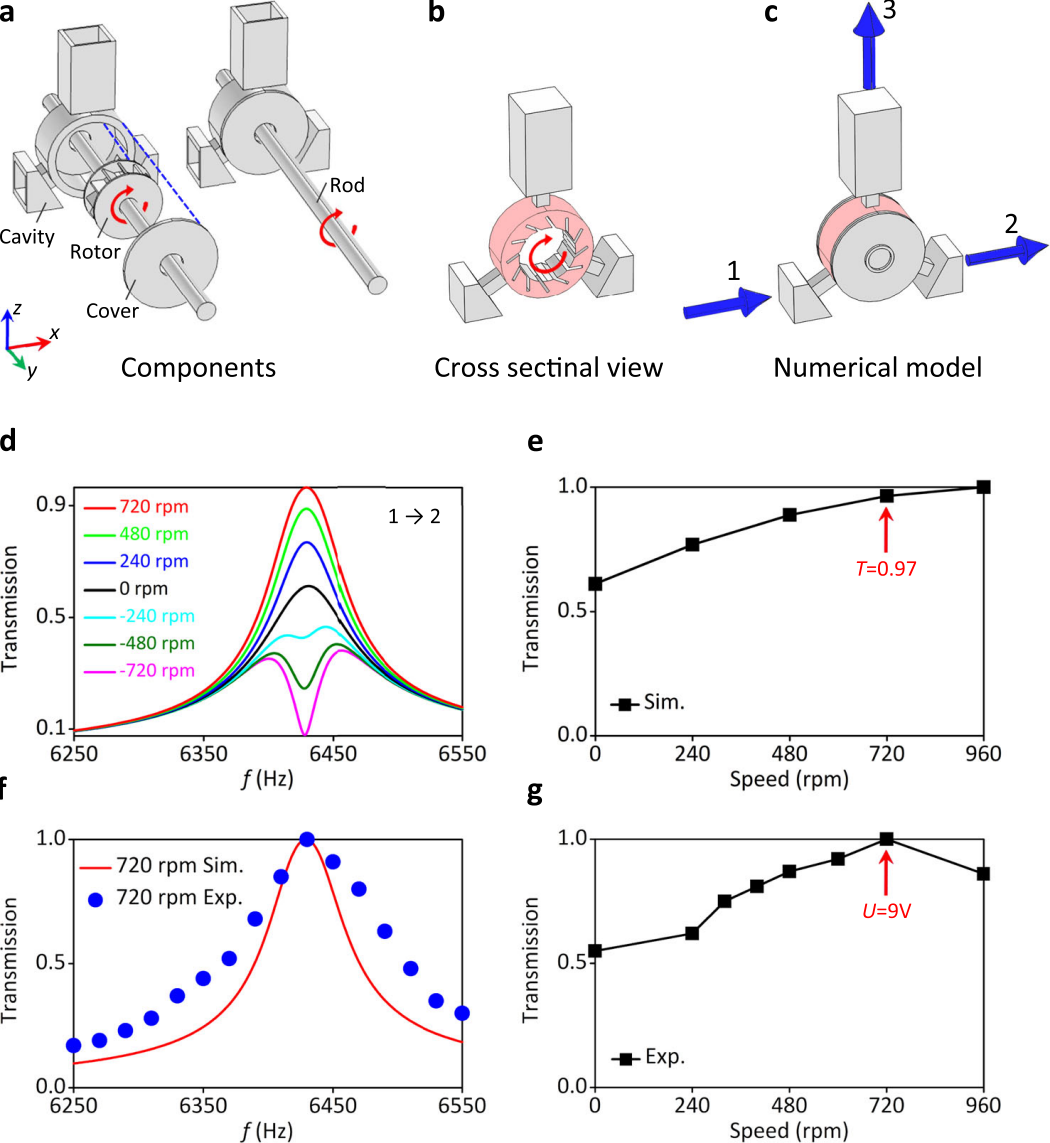
Design of the acoustic circulator. **a** Schematic diagram of the single acoustic circulator consisting of cavity, rotor, cover, and rotating rod as marked in the figure. **b** The cross sectional view of the circular. **c** The physical model for the numerical simulation of the circulator. The three ports (ports 1–3) are marked by blue arrows. **d** The simulated transmissions from port 1 to port 2 for the single circulator with different rotation speeds *n* = ±720 rpm, *n* = ±480 rpm, *n* = ±240 rpm, and *n* = 0 rpm. The maximum and minimum of transmission appear at 6430 Hz with *n* = +720 rpm and *n* = −720 rpm, respectively. **e** The simulated transmission as function of the rotation speed. At the optimized rotation speed *n* = 720 rpm, the transmission is of about 0.97. **f** The simulated (Sim., *n* = 720 rpm) and experimental (Exp., *n* = 720 rpm) transmissions around 6430 Hz. **g** The experimental transmission as function as the rotation speed. The maximum transmission is reached at *n* = 720 rpm, corresponding to the motor voltage of *U* = 9 V.

To break the reciprocity of the acoustic wave propagation, we use an acoustic circulator as sketched in Fig. 2a–c. Acoustic circulators, by their ability to efficiently break reciprocity^32^, have been relevant for the demonstration of acoustic Chern insulators^33^. Figure 2 illustrates the circulator we are proposing here, which consists of cavity, cover, and a rotating rotor driven by motors. A cross sectional view is shown in Fig. 2b consisting of three waveguides and a rotating air medium. The numerical model of the system used to model the experimental setup, is shown in Fig. 2c. The system is purposely designed to take into account the trade-off that has to be made between the necessity to keep the rotor rotating smoothly without friction, which requires sufficiently large air gaps, and on the other hand, preventing acoustic leakage which requires the smallest air gaps possible (see Supplementary Note 1).

The circulator resonator has three ports numbered from 1 to 3 with 120^o^ directional difference, as shown in Fig. 2c. The inner core is rotating to induce an air flow circulating inside the cavity (red part), thus introducing the sought external bias. In this case, the sound wave in the resonator with the flowing air as background medium is determined by the aero-acoustics equation

1 $$-\frac{\rho }{{c}^{2}}i\omega (i\omega \psi +{{{{{\bf{V}}}}}}\cdot \nabla \psi )+\nabla \cdot \left[\rho \nabla \psi -\frac{\rho }{{c}^{2}}(i\omega \psi +{{{{{\bf{V}}}}}}\cdot \nabla \psi ){{{{{\bf{V}}}}}}\right]=0,$$

where *ψ* is the velocity potential, **V** = (*V*_*x*_, *V*_*y*_) is the speed of background air flow. The mass density of air is *ρ* = 1.21 kg/m^3^.

In the single circulator resonator case shown in Fig. 2a–c and when the rotation speed is set at *n* = 720 rpm, nearly total transmission (*T* = 0.97) occurs at 6430 Hz from port 1 to port 2 as one can see in Fig. 2d, e. Since all the acoustic energy is transferred to port 3 and radiates to the free space when the rotation speed is set at *n* = −720 rpm, this leads to no transmission from port 1 to port 2. In other words, port 3 is affecting only one of the propagation directions and not the other. The experimental measurement of a single circulator is shown in Fig. 2f, g. We use electro-motors with tunable voltage within 0–12 V of direct current (DC) with the max rotation speed of *n* = 1000 rpm. The experimental results show that the maximum transmission is reached when we set the rotation speed at approximately 720 rpm (the voltage of the motors are set as *U* = 9 V). We, therefore, choose *n* = 720 rpm (*T* = 0.97) as the optimized rotation speed used in the following simulation to make the comparison with the experimental measurement.

**Fig. 3:**
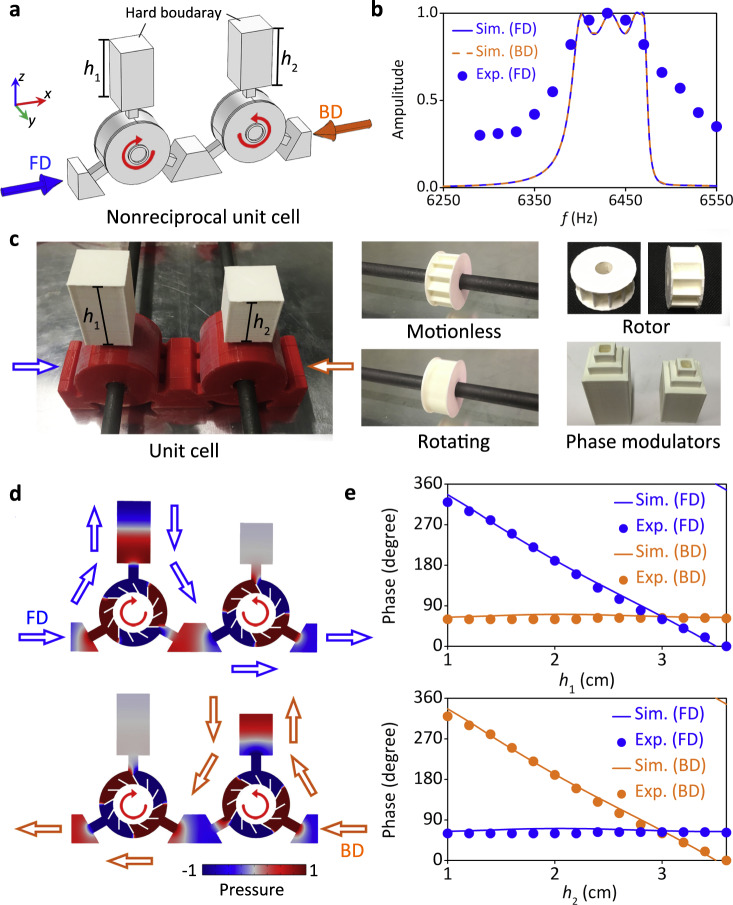
Design of nonreciprocal unit cell. **a** The nonreciprocal unit cell consists of two circulators with opposite rotating inner cores, *viz*., *n*_1_ = −*n*_2_ = 720 rpm, as marked by the red circular arrows. **b** The simulated and experimental (*n*_1_ = −*n*_2_ = 720 rpm) transmissions for FD (forward direction) and BD (backward direction) incidences for the unit cell with *h*_1_ = *h*_2_ = 3 cm. **c** Photograph of the unit cell sample, it consists of 3D printed rotors (white), cavities (red), and reconfigurable phase modulators (white). The blue and orange arrows indicate the FD and BD, respectively. The rotors are nested on carbon rods, which are driven by motors. **d** The acoustic pressure field distributions in the resonators at 6430 Hz for FD and BD incidences, respectively. The arrows indicate the sound propagating paths. **e** The simulated (Sim.) and experimental (Exp.) phase responses at 6430 Hz for FD and BD, by independently changing the parameters of *h*_1_ and *h*_2_, respectively.

An efficient JAM requires total transmission from both sides (FD and BD), the nonreciprocity is restricted to the phase shifts only. This can be achieved by having two circulator resonators 1 and 2 where their inner cores are rotating in opposite direction with respect to each other as shown in Fig. 3a. The rotation speed of the inner cores are *n*_1_ = *−n*_2_ where the subscripts 1 and 2 stand for resonators 1 and 2, respectively. The two ports 3 of the two circulators 1 and 2 are no longer opened to the free space but are closed by rigid backings at a distance *h*_1_ and *h*_2_, respectively. For rotating rotors with *n*_1_ = −*n*_2_ = 720 rpm, the transmission is large in the frequency range from about 6380 to 6490 Hz, with the same amplitude for both FD and BD, while the experimental results show a good agreement with the numerical simulations as illustrated in Fig. 3b. The details of the quality factors (*Q*-factors) and bandwidth for simulations and experiments are shown in the Supplementary Note 2. The detailed analysis of the transmission amplitude is shown in the Supplementary Note 3.

To emphasize the non-reciprocity of the phase shift, we consider the fabricated unit cell sample in Fig. 3c, which consists of 3-D printed rotors (white), cavities (red) and reconfigurable^34^ phase modulators (white). The reconfigurable phase modulators are cuboid grooves with changing heights of *h*_1_ and *h*_2_ for the left and right resonators, respectively. The width of the unit cell is *w* = 1.8 cm. Observing the acoustic pressure field distributions in the resonators at the resonant frequency 6430 Hz in Fig. 3d, one can see that the quadrupolar resonance modes are excited in the cavities. The rotation speed is determined by the resonant mode in the cavity^33^, so we choose the quadrupole mode since its corresponding rotation speed of 720 rpm can be experimentally realized. The sound propagating path in the unit cell is indicated by the arrows in Fig. 3d. Due to the opposite rotating directions in the unit cell and the associated non-reciprocity, the FD wave is coupled to the left phase modulator, while the BD wave is coupled to the right one, leading to the independent and decoupled control of the transmitted phase *φ*_1_ and *φ*_2_. This decoupled phase modulation is validated by both numerical simulation and experimental measurement as delineated in Fig. 3e. The phase *φ*_1_ (*viz*., FD) and *φ*_2_ (*viz*., BD) at 6430 Hz are modulated by independently changing the parameters *h*_1_ and *h*_2_, respectively, within the range of *h*_1/2_ = 1–3.6 cm. Therefore, the JAM device can be conveniently further designed, by providing *h*_1_ and *h*_2_ profiles of the two phase modulators to mimic the desired phase profiles for FD and BD incidences. If the rotation speed is lower than the predesigned one of *n* = 720 rpm, the phase modulations for the two directions will slightly couple. The specific analysis for decoupled and coupled phase modulations can be found in Supplementary Note 4.

**Fig. 4:**
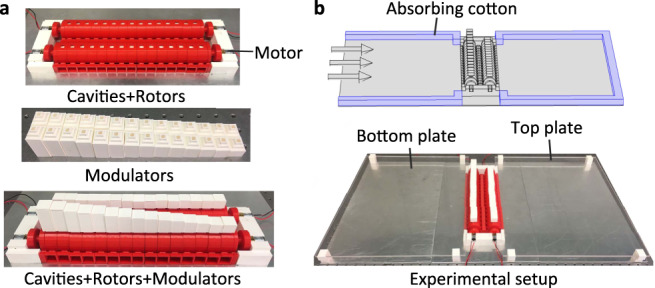
Experiment setup. **a** Photograph of different components of the JAM, including rotors, cavities, and modulators. The array of JAM device consists of 15 unit cells. **b** Schematic diagram and the photograph of the experiment setup.

The experimental fabrications of reported JAM are described in Fig. 4. Figure 4a shows photograph of different components of JAM device to exhibit its fabrication details. The JAM is consisting of 15 reconfigurable unit cells, same as the model used in the simulation. Each unit cell has a width of 1.8 cm, and the whole array has a total width of 28.3 cm (considering the wall thickness), which can be driven by the four motors in experiments. As shown in Fig. 4a, a unit cell is assembled with two cavities, two rotors, and two groove-shape reconfigurable phase modulators. The array is fixed on two carbon rods driven by the four motors. The schematic diagram and the photograph of the experiment setup are shown in Fig. 4b. The performance of the JAM device is measured in a parallel waveguide experimental system^34^. The sound source is a loudspeaker approximately 60 cm away (far field) from the JAM sample to generate plane wave incidence as indicated by the arrows in Fig. 4b. Absorbing cottons are placed at the boundaries of the waveguides to prevent the undesired reflections. The motors are set as 9 V which produce approximately 720 rpm rotation speed with low background noise. The measured signal to noise ratio of the experiment system is 15.2 dB (see Supplementary Note 5). In the experiment, the amplitude of the acoustic field is experimentally measured with one microphone over a rectangular scan region of dimension 24 cm × 48 cm with a step of 2 cm. A second microphone is fixed near the acoustic source, and used as a reference to obtain the phase information.

### Nonreciprocal wavefront manipulations

Based on the numerical and experimental results of the nonreciprocal unit cell, we can design both nonreciprocal^21^ and two-faced^5^ wavefront manipulations.

**Fig. 5:**
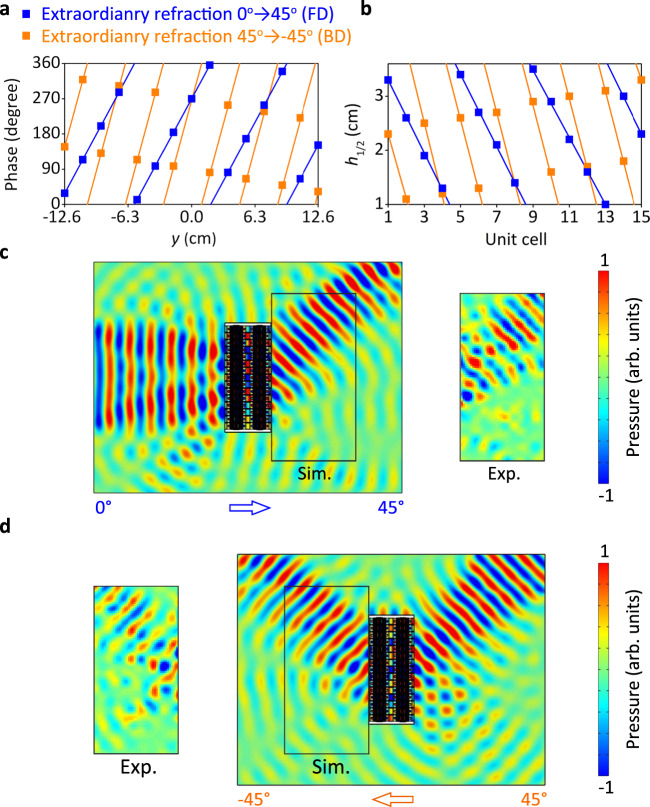
Nonreciprocal acoustic wavefront manipulation for extraordinary refraction. In the FD (forward direction), the normal plane incident wave is converted into an extraordinary refracted plane wave with an angle of 45°. The time-reversed wave of this refracted plane wave is the incident wave in the BD (backward direction) with an angle of 45° which is converted into an extraordinary refracted plane wave with an angle of −45°, which is not the time-reversed wave of the incident one in the forward direction, demonstrating the non-reciprocity of the JAM. **a** The phase profiles along the *y*-direction for the FD and BD, which are *φ*_1_ = 83.3 *y* rad and *φ*_2_ = 166.6 *y* rad, respectively. **b** Corresponding height *h*_1_ and *h*_2_ for the FD and BD, respectively. **c** FD and **d** BD simulated (Sim.) and experimental (Exp.) acoustic pressure distribution at 6430 Hz.

As a functionality of JAM, we first numerically demonstrate the nonreciprocal wavefront manipulation as illustrated in Fig. 5. In the FD, the acoustic wavefront is manipulated from normal plane incidence to 45° refraction angle. To demonstrate the nonreciprocal property of the JAM, the incidence angle in the BD is 45°, which is the time-reversed wave of the transmitted one from the FD. One can clearly see from Fig. 5 that the transmitted wave from the BD is an extraordinary refracted wave with an angle of −45°, which obviously not the time-reversed wave of the incident one from the FD. This demonstrates the nonreciprocal wavefront manipulation. The metascreen is encoded as follows with the phase profiles exposed in Fig. 5a. Based on the generalized Snell’s law, the incident angle *θ*_*i*_ and refraction angle *θ*_*r*_ satisfy the relationship of

2 $$\varphi =k{y}({{{{{\rm{sin}}}}}}\,{\theta }_{r}-\,\sin \,{\theta }_{i})+{C},$$

where *C* is an arbitrary constant, that indicate the initial phase for the center unit cell at *y* = 0 cm. Then, the phase profile of FD is *φ*_1_ = 83.3 *y* + 1.5*π* rad, and the phase profile of BD is *φ*_2_ = 166.6 *y* + 1.5*π* rad, as shown in Fig. 5a. The optimization of the constant *C* in the predesigned phase profile can be found in Supplementary Note 6.

The step of the discrete point in Fig. 5a is 1.8 cm, which is equal to the unit cell width. This width corresponds to about *λ*/3 at the working frequency. This means that there are three discrete phase points in one wavelength in the transverse direction of the JAM. A numerical simulation with higher transverse resolution of the metasurface phase profile with a unit cell width of 1 cm corresponding to *λ*/5 at the working frequency is shown in the Supplementary Note 7. Based on the discrete phase profile of *φ*_1_ and *φ*_2_ as illustrated in Fig. 5a, we achieve the desired values of *h*_1_ and *h*_2_. The calculated discrete *h*_1_ and *h*_2_ for FD and BD are shown in Fig. 5b. By designing the array with these parameters, we obtain the JAM array with a total width of 27 cm. We choose using 15 unit cells to build the whole array in simulation, because this number is large enough to generate highly efficient beam steering. The simulated acoustic pressure distributions at 6430 Hz is shown in Fig. 5c, d for FD and BD, respectively. The FD incident wave is redirected to 45^o^ angle at Region 2, after interaction with the JAM. On the contrary, The BD incident wave is transmitted with an inversed direction of 45^o^ through the JAM to Region 1 with −45^o^ angle. The experimental results are shown in the inset, that agree well with the simulated results, that shows a non-reciprocal process. The nonreciprocal wavefront manipulation is robust against variation of the rotation speed (see Supplementary Note 4).

**Fig. 6:**
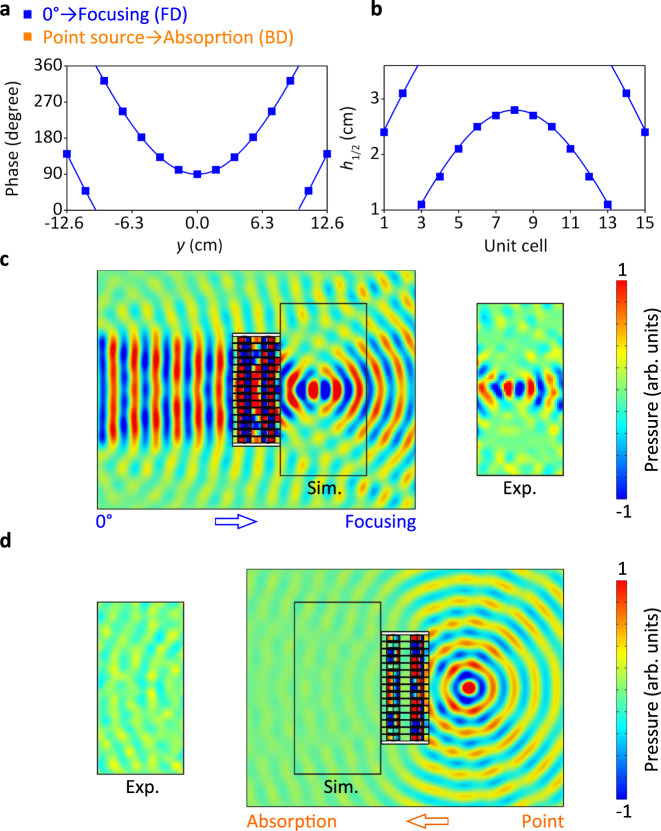
Nonreciprocal acoustic wavefront manipulation for acoustic focusing. In the FD (forward direction), the normal plane incident wave is focused in a focal spot situated at *x* = 0 and *y* = 0.1 m. The time-reversed wave of this focal spot is the incident wave in the BD (backward direction), which is a point source at the same position as the focal spot. This gets totally absorbed through leakage losses, which is not the time-reverse of the incident wave from the FD demonstrating the non-reciprocity of the JAM. **a** Phase profiles along the *y*-direction for the FD, which is *φ*_1_ = 117.8((*x* − 0.1)^2^ + *y*^2^)^0.5^ − 11.78 + *π*/2 rad. For the BD, the modulators have open boundaries giving total absorption through leakage losses. **b** Corresponding height *h*_1_ for the FD. **c** FD and **d** BD simulated (Sim.) and experimental (Exp.) acoustic pressure distribution at 6430 Hz.

As a second example and demonstration of the nonreciprocal wavefront manipulation, we show in Fig. 6c that the acoustic wavefront from the FD is manipulated from normal plane incidence to a focal spot. The incident wave from the BD is the time-reversed wave of the focal spot which is a point source situated at the same position. On the other side, one can observe from Fig. 6d that the acoustic wave coming from the BD is totally absorbed through leakage losses in the modulators, which is clearly not the time-reversed wave of the incident wave in the FD. This demonstrates the nonreciprocal wavefront manipulation. The metascreen is encoded as follows with the phase profiles exposed in Fig. 6a. The phase profile for acoustic focusing can be obtained by

3 $$\varphi =k\left[\sqrt{{(x-{x}_{0})}^{2}+{({y}-{y}_{0})}^{2}}-\sqrt{{{x}_{0}}^{2}+{{y}_{0}}^{2}}\right]+{C},$$

where (*x*_0_, *y*_0_) is the predesigned focal point. Then, the phase profile of FD for acoustic focusing at coordinate (0.1, 0) is *φ*_1_ = 117.8((*x* − 0.1)^2^ + *y*^2^)^0.5^ − 11.78 + *π*/2 rad, as the curve and the discrete points shown in Fig. 6a. The corresponding *h*_1_ values for 15 unit cells are displayed in Fig. 6b, extracted from the data in Figs. 3e and 6a. For BD case, we use the modulators with opened boundaries to realize total sound absorption, taking advantage of the leakages at the opened boundaries^17^. Therefore, the effective *h*_2_ value is *h*_2_ → ∞. The simulated acoustic pressure distributions at 6430 Hz is shown in Fig. 6c. Different from Fig. 5c, in this simulation, the plane wave radiation boundaries instead of hard boundaries are applied at the right modulators to mimic the opened boundaries. For FD case, the incident wave is focused at the coordinate (0.1, 0) at Region 2. For the BD case, the incident waves from the point source are transmitted to the right modulators and get totally radiated from the opened boundaries, leading to the effective sound absorption via sound leakage phenomenon^17^.

### Two-faced wavefront manipulations

**Fig. 7:**
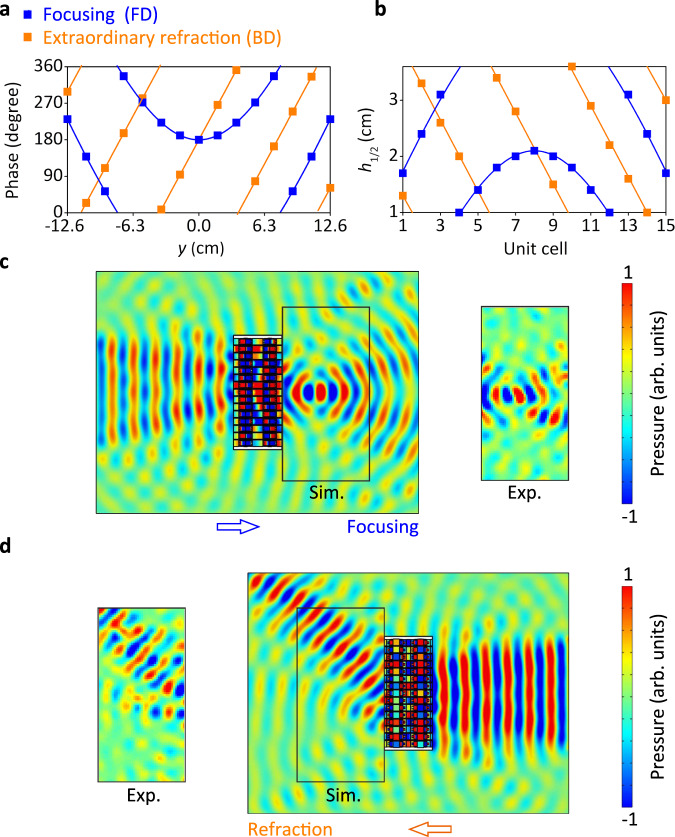
Two-faced wavefront manipulation, encoding focusing for FD (forward direction), and extraordinary refraction for BD (backward direction). **a** The phase profiles along *y*-direction of acoustic focusing for FD and extraordinary refraction for BD are *φ*_1_ = 117.8((*x* − 0.1)^2^ + *y*^2^)^0.5^ − 11.78 + *π* rad, and *φ*_2_ = 83.3 *y* + *π* rad, respectively. **b** Corresponding *h*_1_ and *h*_2_ values for FD and BD, respectively. **c**, **d** Corresponding simulated (sim.) and experimental (exp.) acoustic pressure distributions at 6430 Hz for PD in (**c**) and BD in (**d**).

Compared with above nonreciprocal manipulations, the two-faced manipulation that encodes two kind of beam steering into one acoustic metascreen, is an advanced functionality of the JAM with real added values. The first design is a combination of acoustic focusing and extraordinary refraction in JAM. Based on Eqs. (2) and (3), the phase profiles for FD and BD are calculated as *φ*_1_ = 117.8((*x* − 0.1)^2^ + *y*^2^)^0.5^ − 11.78 + *π* rad, and *φ*_2_ = 83.3 *y* + π rad, as shown in Fig. 7a. We get the parameter profiles of the phase modulators in Fig. 7b for satisfying two entirely different predesigned phase profile *φ*_1_ and *φ*_2_. The corresponding simulated results for two-faced manipulation are shown in Fig. 7c, d. The acoustic pressure distributions at 6430 Hz show that the acoustic focusing and extraordinary refraction are realized for FD and BD, respectively.

The second example, we propose here, is the combination of acoustic diffusion and beam splitting. The acoustic diffusion has the opposite^34^ phase profile to the acoustic focusing, expressed as

4 $$\varphi ={k}\left[\sqrt{{{x}_{0}}^{2}+{{y}_{0}}^{2}}-\sqrt{{(x-{x}_{0})}^{2}+{({y}-{y}_{0})}^{2}}\right]+{C},$$

The phase profile for acoustic splitting is a piecewise function for *y* ≤ 0 and *y* > 0 regions, respectively. For a symmetrical splitting, the phase profile is an even function, expressed as

5 $$\begin{array}{c}\varphi =k{y}(\sin \,{\theta }_{r}-{{{{{\rm{sin}}}}}}\,{\theta }_{i})+{C},y\le 0\\ \varphi =k{y}(\sin \,{\theta }_{i}-{{{{{\rm{sin}}}}}}\,{\theta }_{r})+{C},y \, > \, 0\end{array}$$

**Fig. 8:**
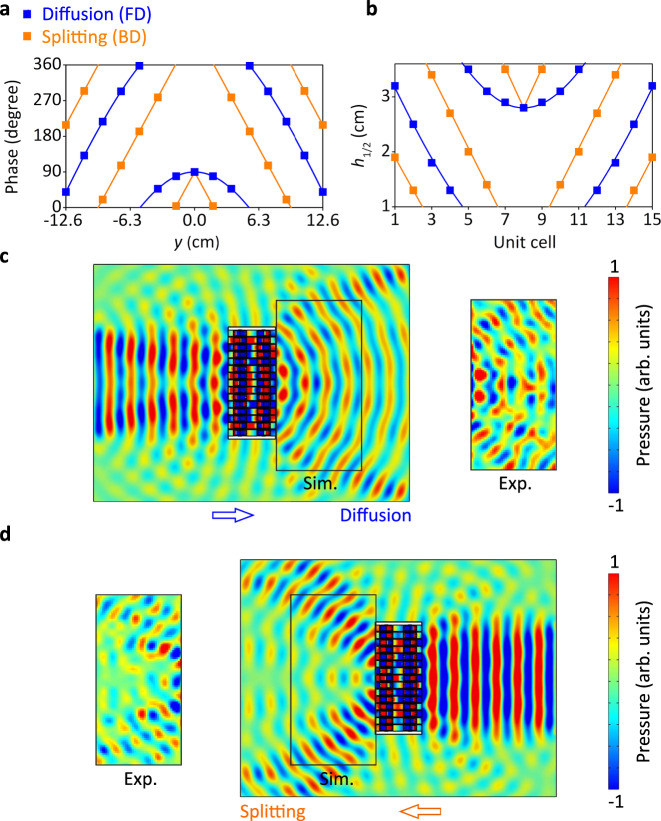
Two-faced wavefront manipulation, encoding diffusion for FD (forward direction), and splitting for BD (backward direction). **a** Phase profiles of acoustic diffusion for FD and beam splitting for BD are *φ*_1_ = 11.78 − 117.8((*x* − 0.1)^2^ + *y*^2^)^0.5^ + *π*/2 rad and *φ*_2_ = −83.3 | *y* | + *π*/2 rad, respectively. **b** Corresponding *h*_1_ and *h*_2_ values for FD and BD, respectively. **c**, **d** Corresponding simulated (sim.) and experimental (exp.) acoustic pressure distributions at 6430 Hz for PD in (**c**) and BD in (**d**).

Thus, the phase profiles for acoustic diffusion and beam splitting are *φ*_1_ = 11.78 − 117.8((*x* − 0.1)^2^ + *y*^2^)^0.5^ + *π*/2 rad and *φ*_2_ = −83.3 | *y* | + *π*/2 rad, respectively, as shown in Fig. 8a. Following the same design process, we get the *h*_1_ and *h*_2_ values displayed in Fig. 8b, corresponding to the two phase profiles. The simulated acoustic pressure distributions for acoustic diffusion and beam splitting at 6430 Hz are shown in Fig. 8c, d. Regarding the acoustic diffusion, the acoustic pressure amplitudes are relatively balanced for different directional components^35^. For the acoustic splitting, the simulated normalized directional coefficients have two peaks at ±45°^36,37^. The detailed analysis of simulated and experimental results can be found in the Supplementary Note 8. The above two groups of results suggest that the JAM can realize decoupled wavefront modulations for the two opposite incidences with high efficiency and low cross-talk effect, benefiting from the highly nonreciprocal mechanism.

## Discussion

We have introduced and demonstrated the concept of Janus acoustic metascreen realizing both nonreciprocal and two-faced wavefront manipulations from two opposite incidences. The unprecedented features of two-faced wave modulations require nonreciprocal phase modulations of the acoustic unit cell. We have used a time-varying acoustic resonant unit cell with rotating inner core to achieve high nonreciprocity, leading to decoupled phase modulations for forward and backward directions by independently changing two structural parameters. We have experimentally fabricated reconfigurable phase modulating unit cells, and therefore, a series of Janus acoustic metascreens producing two-faced functionalities have been numerically and experimentally demonstrated. Our finding opens a new way for nonreciprocal and Janus acoustic meta-devices for myriad of multifunctional and compact systems as well as related applications.

## Methods

### Numerical simulations

The simulations are performed using the commercial finite element analysis software, COMSOL Multiphysics 5.6a with the module of “Aeroacoustics, Linearized Potential Flow, Frequency Domain”. In simulations, we set the speed of air flow in the resonators. The considered mass density and the sound speed of background medium air are *ρ* = 1.21 kg/m^3^ and *c* = 343 m/s, respectively. The solid materials in the unit cells are set as sound hard boundaries. For the simulations in Fig. 1, the top boundaries of port 3 are set as plane wave radiation boundaries. For other simulations throughout the paper, the top boundaries of port 3 are set as sound hard boundaries.

### Sample fabrications

The samples are fabricated using a commercial extrusion-based 3D printer (Ultimaker 3) and the material of poly-lactic acid, with the mass density *ρ*_1_ = 1250 kg/m^3^, Young’s modulus *E*_1_ = 3.2 × 10^9^ Pa, Poisson’s ratio *ν*_1_ = 0.35. The size of the whole device is 40 cm × 12 cm × 7.5 cm with an optimized configuration which corresponds to 7.5*λ* × 2.3*λ* × 1.4*λ* at the working frequency of 6430 Hz, thus being on the order of magnitude of the wavelength. The width of the array is 40 cm including the rotors at two sides. The length in the direction of propagation of the acoustic waves is 12 cm. The maximal height is 7.5 cm considering the highest phase modulator.

### Experimental measurements

The experiments are conducted in a lab-made plate waveguide system^33^. The size of two pair of plexiglass plates is 0.5 m × 0.4 m at both sides (Regions 1 and 2) of the JAM as shown in Fig. 6(b). The distance between upper and bottom plates is 2 cm. The electro-motors are operated under 9 V (DC) for generate 720 rpm rotation speed. A 10-cm-diameter loudspeaker is used as the source and placed at a distance 60 cm (far field) from the sample, to generate plane wave incidence. Absorbing cottons are placed at the boundaries of the waveguides to prevent the undesired reflections. In experiment, two 1/8-in.-diameter Brüel&Kjær type-4138-A-015 microphones and Brüel&Kjær PULSE Type 3160 are used for measurement. The amplitude of the acoustic field is measured with one microphone over a rectangular scan region of dimension 24 cm × 48 cm with a step of 2 cm. A second microphone is fixed near the acoustic source and is used as a reference to obtain the phase information.

## Supplementary information

Supplementary Information

## Data Availability

All relevant data that support the findings of this study are available from the corresponding authors upon reasonable request.

## Code availability

The code used for the analyses will be made available upon e-mail request to the corresponding author.
