# Supplementary material for: Janus acoustic metascreen with nonreciprocal and reconfigurable phase modulations
Source: Nat Commun. 2021 Dec 6;12:7089. doi: 10.1038/s41467-021-27403-4 (PMC8648919; doi:10.1038/s41467-021-27403-4)
Supplement: Supplementary file 1 — Supplementary Information [file 41467_2021_27403_MOESM1_ESM.pdf]

Supplementary Information of "Janus acoustic metascreen with nonreciprocal and reconfigurable phase modulations"

Yifan Zhu[1*], Liyun Cao[1], Aurélien Merkel[1], Shi-Wang Fan[1], Brice Vincent[1] and Badreddine Assouar[1*]

[1]Université de Lorraine, CNRS, Institut Jean Lamour, Nancy, 54000, France.

Corresponding authors:

*yifan.zhu@univ-lorraine.fr

*badreddine.assouar@univ-lorraine.fr

Note 1. Details of the system.

Note 2. Operational frequency bandwidth in simulations and experiments.

Note 3. Detailed analysis of the transmission amplitude.

Note 4. Phase modulation and wavefront manipulation against the rotation speed.

Note 5. Background noises and signal-to-noise ratio.

Note 6. Optimization of predesigned phase profile.

Note 7. Transverse resolution of the metascreen phase profile.

Note 8. Detailed analysis of simulated and experimental results.

Supplementary Note 1. Details of the system.

The details of the system are shown in Supplementary Figure 1(a). The width of air part in the rotor is w_1=12 mm. The wall thickness of the rotor is t_0=1 mm. The width of the rotor is $w_2=w_1+2t_0$=14 mm. The transverse width of the air gap in the cavity is t_1=1 mm. The inner width of the cavity is $w_3=w_2+2t_1$=16 mm. The wall thickness of the cavity is t_2=2 mm. The outer width of the cavity is $w_4=w_3+t_2$ =18 mm. (equal to the width of unit cell w =18 mm). The area of the waveguide (including Ports 1-3, Port 3 is phase modulator) is S=$w_3\times w_3$=16×16 mm^2. The area of the waveguide considering the wall thickness is $S_1=w_4\times w_5$=18×20 mm^2

The radius of the rotor is R_0=15.2 mm. The inner radius of the cavity is R_1=16.4 mm. So the radial air gap is $t_3= R_1-R_0$=1.2 mm. The outer radius of the cavity and the cover is R_2=20 mm. The radiuses of the rod and rotor are both r_0=4 mm, insuring that the rotor can be driven by the rod. The radius of the hole on the cavity is r_1=5 mm. So the radial air gap between the rod and the hole is $t_4= r_1-r_0$=1 mm. The transverse gap between the rod and the hole is $t_2/2$=1 mm. The width of the narrow square waveguide is w_0=4.6 mm. Above sizes show that the air gaps are always within 1 mm to 1.2 mm to ensure the rotation of cavity with rod without frictions.

A lateral view is shown in Supplementary Figure 1(b). The transverse size of the unit cell is w=18 mm. Two covers are placed at the both side of the cavity with the thickness of 7 mm and 6 mm, respectively. So for the unit cell experiment, the transverse size is 7+18+6=31 mm. For the array, the total transverse size without (with) motor part is 7+18×15+6=283 mm (46+283+46=375 mm).

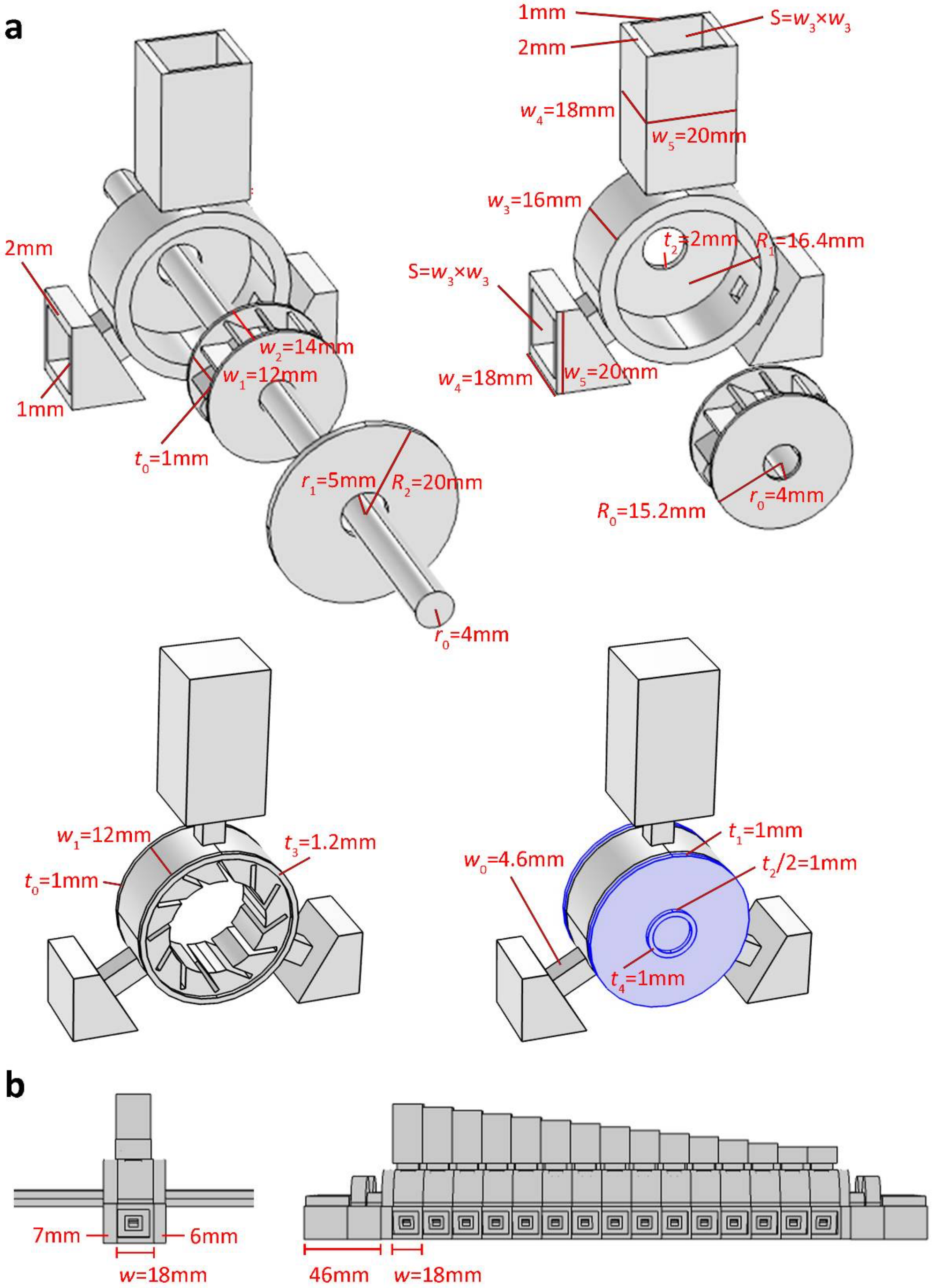

Supplementary Figure 1. Details of the system. (**a**) The structural parameters of the assembled structures. (**b**) The structural parameters in the array.

Supplementary Note 2. Operational frequency bandwidth in simulations and experiments.

As can be seen in Fig. 3(d) in the main text, the non-reciprocity is obtained around the frequency of the quadrupolar resonance mode of the circulators. Therefore the operational frequency bandwidth is limited by the resonance curves in transmission. We estimate from the transmission curves in Supplementary Figure 2(a) [same as Fig. 3(b) in the main text] the frequency bandwidth Δf at $T > 1/\sqrt{2}$ from which is derived the Q-factor $Q=f/\Delta f$ where f is the resonance frequency. By considering the unit cell formed by two circulators, the Q-factor depends on the geometrical parameter w_0, which can be increased in order to broaden the operational frequency bandwidth as shown in Supplementary Figures 2(b) and (c). The rotation speed at which the transmission of the unit cell is maximum also increases while increasing this parameter w_0.

Considering experimental limitations, we set the maximum rotation speed at 720 rpm and the corresponding waveguide width is w_0=4.6 mm as shown in Supplementary Figure 2(d). For the unit cell, the Q-factor from the numerical simulations is Q_{sim}=80.4 with the corresponding bandwidth Δf=80 Hz (6393-6473 Hz). In experiments, the Q-factors are Q_{exp}=58.5 and with the corresponding bandwidth Δf=110 Hz (approximately 6380-6490 Hz).

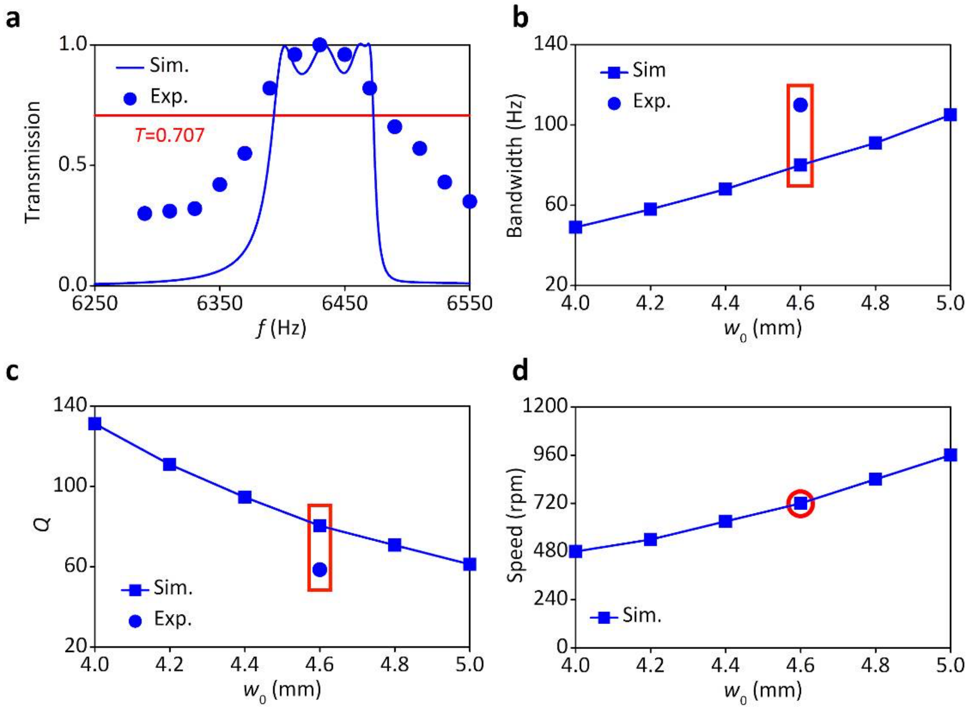

Supplementary Figure 2. Details of the bandwidth of unit cell. (**a**) Simulated (Sim.) and experimental (Exp.) transmission for the unit cell. Dependence on the geometrical parameter w_0 (**b**) of the frequency bandwidth Δf and (**c**) of the *Q*-factors. The corresponding experimental values are plotted for the case w_0=4.6 mm. (**d**) Dependence on w_0 of the rotation speed at which the transmission of the unit cell is maximum.

Supplementary Note 3. Detailed analysis of the transmission amplitude.

In Fig. 3(b) of the main text, the simulated transmission amplitude obtained at h_1=h_2=3 cm, not considering the visco-thermal effect. In the simulations of Supplementary Figure 3, we study the influences of heights of the both cavities h_1 and h_2, visco-thermal losses, and rotor leakage.

Supplementary Figures 3(a-d) show the transmission of the unit cell with h_1=1, 2, 3, 4 cm and h_2=3 cm, for 720, 480, 0 rpm, respectively. The resonant frequency 6430 Hz is marked by blue dashed lines. The results show that the transmission amplitude around 6430 Hz is mainly decided by the rotation speed, and not so relevant to the h_1 and h_2. The simulated results show that if the rotation speed drops to 480 rpm, the transmission amplitude is near 0.8.

Supplementary Figure 3(e) shows the simulated transmission amplitude of original numerical model in main text, and the model considering the rotor leakage of the air gap. We set periodic boundary conditions that mimic the unit cell in the array in original model. We set the air gap connected to outside space in the model considering the rotor leakage. The two numerical models under n=720 rpm has similar transmission amplitude as shown in Supplementary Figure 3(e) demonstrating that the sound leakage in the unit cell is very small.

Supplementary Figure 3(f) shows the transmission amplitude for n=0 rpm simulated in the modules of "Aeroacoustics, Linearized Potential Flow, Frequency Domain" and "Acoustic-Thermoviscous, Acoustic Interaction, Frequency Domain", respectively. The latter considering visco-thermal effect is very close to the former, showing the dissipation of the unit cell is trivial.

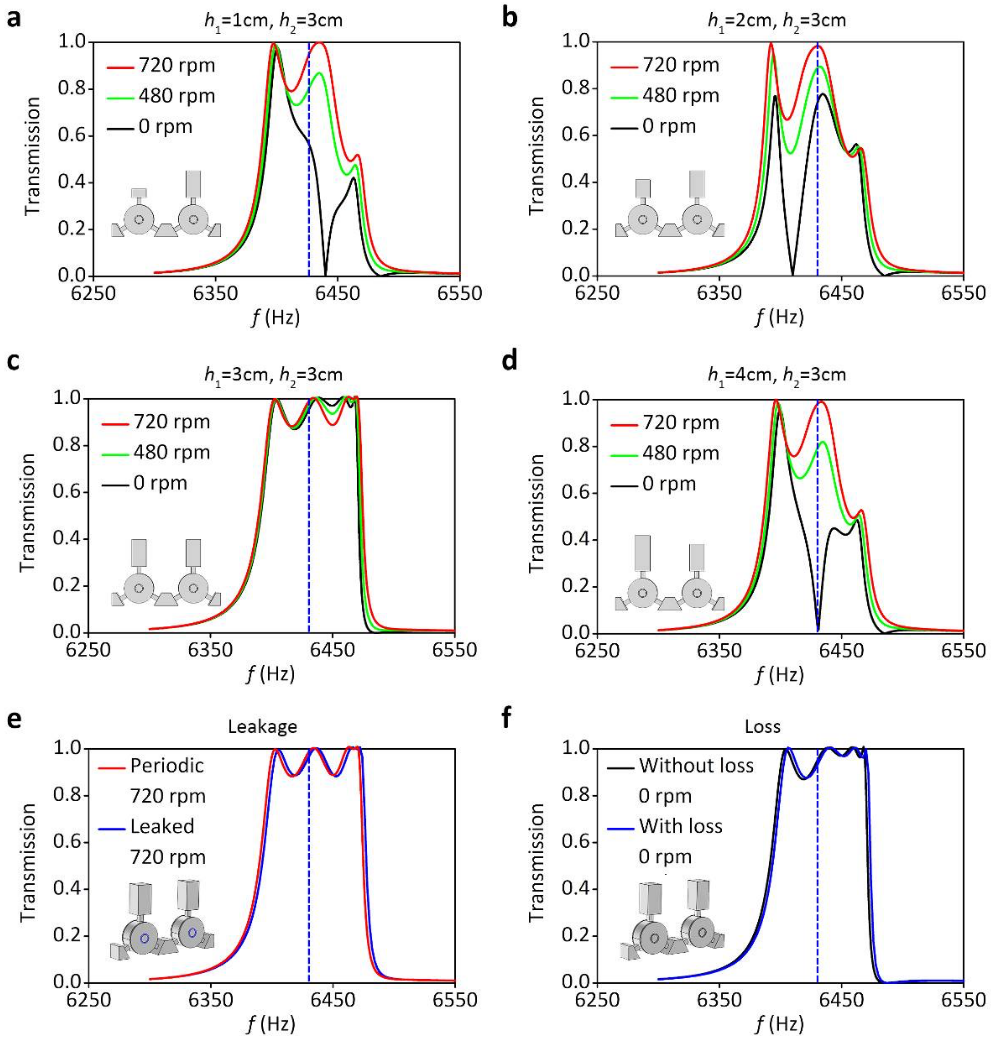

Supplementary Figure 3. Parameter dependence for transmission amplitude. (**a-d**) Transmission of the unit cell with h_1=1, 2, 3, 4 cm and h_2=3 cm, for 720, 480, 0 rpm, respectively. The resonant frequency 6430 Hz is marked by blue dashed lines. (**e**) The original numerical model with periodic boundary condition and the leaked model that connecting air gap to outside space. (**f**) The numerical models without and with visco-thermal effects.

Supplementary Note 4. Phase modulation and wavefront manipulation against the rotation speed.

Numerical simulations have been performed to evaluate the robustness of the phase modulation and wavefront manipulation of the JAM when the rotation speed of the rotors varies with n=720, 480, 240, 120, 60 and 0 rpm. As shown in Supplementary Figure 4(a), when the rotation speed is set at n=720 rpm, only the phase φ_1 is modulated by varying the height h_1 with fixed h_2=3 cm and the phase φ_2 remains constant. Similar to Fig. 3(e) in the main text, if we interchange h_1 and h_2, that is varying height h_2 with fixed h_1=3 cm, the curve of φ_1 and φ_2 will be interchanged, that is, φ_2 is modulated by h_2 and the phase φ_1 remains constant (not shown in Supplementary Figure 4). Thus, at n=720 rpm, the modulation of the phases φ_1 and φ_2 are completely decoupled.

When we decrease the rotation speed into n=480, 240, 120, 60 rpm, the phase φ_1 is modulated by varying the height h_1 as shown in Supplementary Figure 4(a) and the phase φ_2 is varying as well. This means that the modulation of φ_1 and φ_2 is not decoupled anymore. When the rotation speed is decreasing, the phase difference between φ_1 and φ_2 is decreasing, means the effect of nonreciprocity is decreasing. When the rotation speed is n=0 rpm, we get a reciprocal phase modulation with φ_1=φ_2 as shown in the last sub-figure of Supplementary Figure 4(a).

It is noted that for n=0 rpm, the phase modulation of φ_1(=φ_2) is constrained in the range from 0 to 180°, while for n>0 rpm, the phase φ_1 shows an extended range from 0 to 360. For n=0 rpm, there is a phase jump around h_1=1.36 cm. At h_1=1.35 cm, φ_1=0°, and at h_1=1.36 cm, φ_1=180°. This phase jump can be regarded as increasing φ_1(=φ_2) from 0° to 180° (orange line), or decreasing φ_2(=φ_1) from 360° to 180° (blue line). To have a better

understanding of this phase jump, we show the dependence of φ_1 (φ_2) on h_1 (h_2) for different rotation speeds *n* in the left (right) figure in Supplementary Figure 4(b). Considering the evolution of the phases φ_1 and φ_2 when decreasing *n* from 720 rpm to 0 rpm, the different phase jumps (360° to 180° and 0° to 180° for φ_1 and φ_2, respectively) around h_1=1.36 cm for n=0 rpm (purple lines) seems consistent with the curves observed when $n \neq 0$.

Supplementary Figure 4(c) shows the relationship between φ_1/φ_2 and rotating speed (changing from 720 to 0 rpm), for fixed values of h_1=1.5 cm, h_2=3 cm. For large rotation speed, the values of φ_1 and φ_2 are quite different, demonstrating large effect of nonreciprocity. When the rotating speed is n=0 rpm, we have a reciprocal phase modulation with $\varphi_1=\varphi_2$. Similarly, Supplementary Figure 4(d) shows the relationship between φ_1/φ_2 and rotating speed (changing from 720 to 0 rpm) for fixed values of h_1=1.36 cm, h_2=3 cm. Observing Supplementary Figures 4(c-d), the values of φ_1 and φ_2 are not linearly varying with rotation speeds *n.* They vary slowly when *n* is large, but vary rapidly when *n* is small. So we denote a flat region within 720-240 rpm marked by grey color, in which the background flow has very little impact on φ_1 and φ_2. This feature leads to high robustness of phase modulation and wavefront manipulation against rotation speed, which will be demonstrated in wavefront manipulation later.

To further study the phase modulation against the rotation speed, we shows the acoustic pressure distribution in the unit cell for different rotation speeds of n=720, 480, 240, 120, 60, 0 rpm, respectively, in Supplementary Figures 4(e-f). In Supplementary Figure 4(e), we set h_1=1.5 cm, h_2=3 cm. For n=720 rpm, phase modulator 2 of the unit cell has lowest acoustic pressure distribution that means the incident wave is coupled

with phase modulator 1 and nearly decoupled with phase modulator 2 at highest rotation speed. In Supplementary Figure 4(f), we set h_1=1.36 cm, h_2=3 cm. In this case, the acoustic pressure in phase modulator 2 is always zero. The acoustic pressure distribution for phase jump is shown in the last figure that the acoustic pressure in the second circular is all zero, that means this phase jump (h_1=1.36 cm, h_2=3 cm, n=0 rpm) is corresponding to zero transmission (T=0).

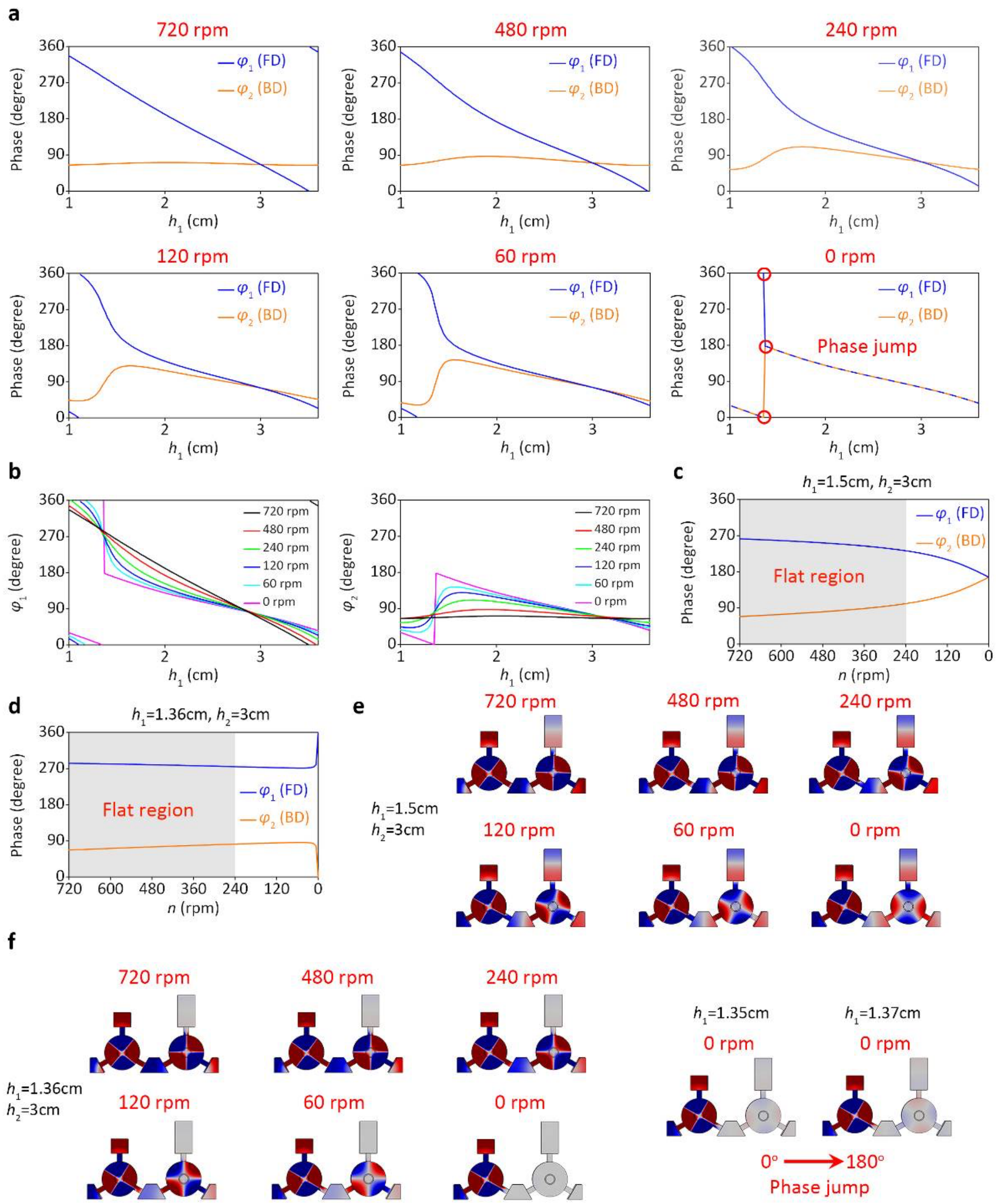

Supplementary Figure 4. Simulated phase responses with different rotating speed. (**a-b**) Simulated phase responses φ_1 and φ_2 at 6430 Hz for FD (forward direction) and BD (backward direction), by independently varying the parameter h_1 (h_2 is fixed as h_2=3 cm), with the rotating speed of n=720, 480, 240, 120, 60, 0 rpm. A phase jump is marked in the figure for n=0 rpm. (**c**) For fixed values of h_1=1.5 cm, h_2=3 cm, the relationship between φ_1/φ_2 and rotating speed (varying from 720 to 0 rpm) (**d**) For fixed values of h_1=1.36 cm, h_2=3 cm, the relationship between φ_1/φ_2 and rotating speed (changing from 720 to 0 rpm). (**e**) For fixed values of h_1=1.5 cm, h_2=3 cm, the acoustic pressure distribution in the unit cell for the rotating speed of n=720, 480, 240, 120, 60, 0 rpm. (**f**) For fixed values of h_1=1.36 cm, h_2=3 cm, the acoustic pressure distribution in the unit cell for the rotating speed of n=720, 480, 240, 120, 60, 0 rpm. The acoustic pressure distribution in the unit cell for the phase jump around h_1=1.36 cm.

In order to study the robustness of wavefront manipulation against rotation speed, we compared the simulated results for wavefront manipulation of the JAM with the rotation speed n=720, 480, 240, 120, 60, 0 rpm, respectively. We take the same case (nonreciprocal extraordinary refraction) as in Fig. 5 of the main text. As can be seen in Supplementary Figure 5, lowering the rotation speed up to 1/3 of the initial one deteriorates the wavefront manipulation without showing dramatic effects, because the flat region of 720-240 rpm marked in Supplementary Figures 4(c-d) has relatively smaller phase difference. In conclusion, the implementation of the JAM is robust against variation of the rotation speed.

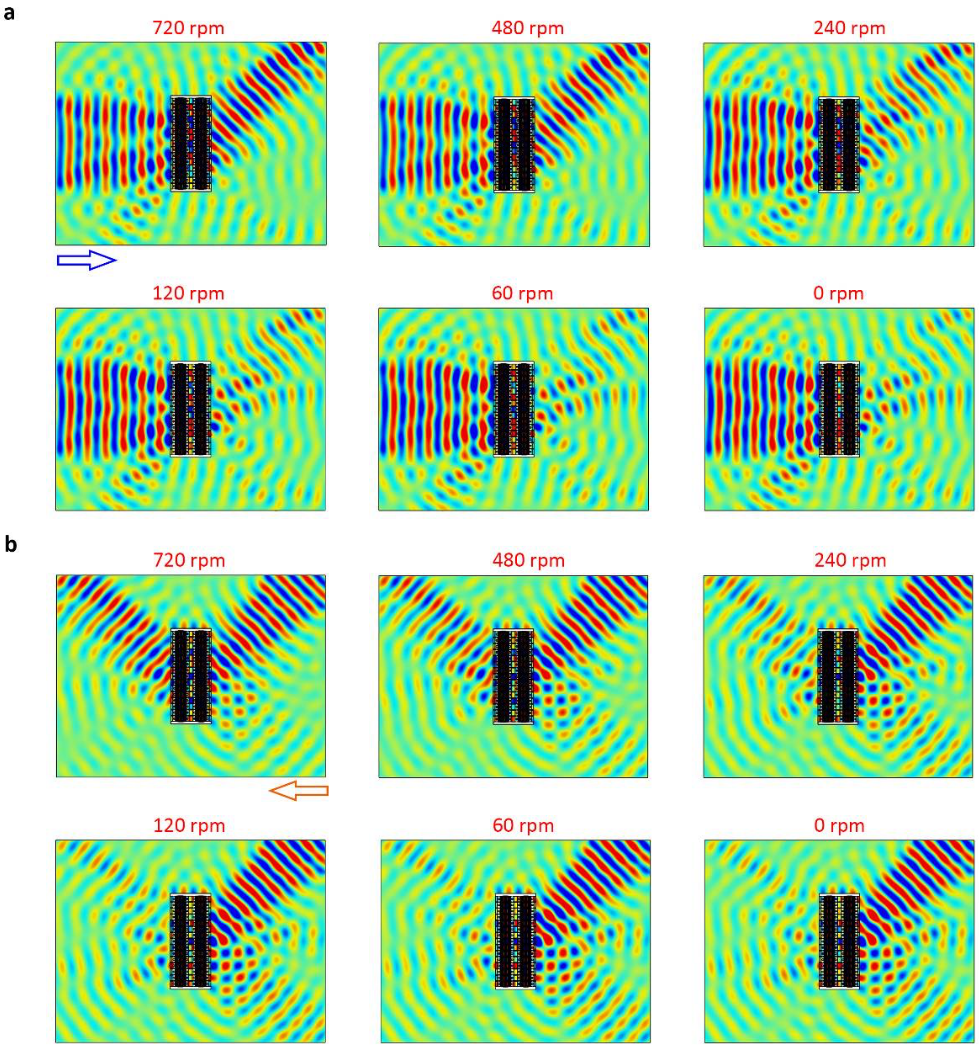

Supplementary Figure 5. Simulated nonreciprocal wavefront manipulations with different rotating speed. Simulated nonreciprocal extraordinary refraction with the rotating speed of n=720, 480, 240, 120, 60, 0 rpm, respectively, for forward direction in **a**, and backward direction in **b**.

Supplementary Note 5. Background noises and signal-to-noise ratio.

We choose "Constant Percentage Bandwidth Analyzer" (CPB) in Brüel&Kjær software to measure the background noise within broadband frequency range, that is from 125 Hz to 20000 Hz with a step of 1/3 octaves. The average sound pressure amplitudes within every 1/3 octaves are shown in Supplementary Figure 6. Supplementary Figures 6(a-b) show the background noises with and without sound source, respectively. The experimental working bandwidth from about 6380 Hz to 6490 Hz are corresponding to the 1/3 octaves bandwidth with the center frequency of 6300Hz, and when the rotation speed is set at 720 rpm, the normalized noise pressure level is measured at L_{noise}= 18.7 dB. When the source is turned on, the normalized acoustic pressure level is measured at L_{ac}=33.5 dB. Thus, the signal to noise ratio is 15.2 dB, which is large enough to ensure consistent measurements.

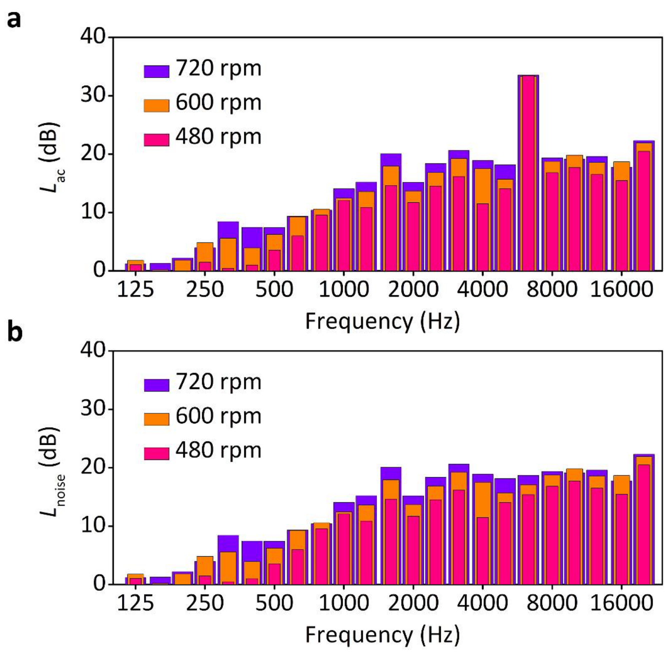

Supplementary Figure 6. Background noises. (**a**) Background noises with sound source for high rotating speed for the rotation speeds of 720, 600, and 480 rpm. (**b**) Background noises without sound source for high rotating speed for the rotation speeds of 720, 600, and 480 rpm.

Supplementary Note 6. The optimization of predesigned phase profile.

In our design, the expression for extraordinary refraction, acoustic focusing, acoustic diffusion and bean splitting are shown in Eqs. 1-4.

$$\varphi = ky(\sin\theta_r - \sin\theta_i) + C, \qquad (1)$$

$$\varphi = k\left[\sqrt{(x-x_0)^2+(y-y_0)^2} - \sqrt{x_0^2+y_0^2}\right] + C, \qquad (2)$$

$$\varphi = k\left[\sqrt{x_0^2+y_0^2} - \sqrt{(x-x_0)^2+(y-y_0)^2}\right] + C, \qquad (3)$$

$$\begin{aligned}\varphi &= ky(\sin\theta_r - \sin\theta_i) + C,\ y \le 0 \\ \varphi &= ky(\sin\theta_i - \sin\theta_r) + C,\ y > 0\end{aligned} \qquad (4)$$

where C is an arbitrary constant that means the phase of the unit cell of the center of the metascreen array (y=0 cm). We test C=0, 90, 180, 270 degrees, respectively, in array simulations, and find the best C values with highest efficiency. We finally get the optimized initial phases for the designs in Figs. 5-8 in the main text. The four C values are 270, 90, 180, 90 degrees, respectively, as shown in Supplementary Figure 7. We therefore get the four pairs of phase profiles for Figs. 5-8 in the main text, as follow

1. φ_1=83.3y+1.5π rad, φ_2=166.6y+1.5π rad.
2. φ_1=117.8$((x\text{-}0.1)^2+y^2)^{0.5}$-11.78+π/2 rad, N/A.
3. φ_1=117.8$((x\text{-}0.1)^2+y^2)^{0.5}$-11.78+π rad, φ_2=83.3y+π rad.
4. φ_1=11.78-117.8$((x\text{-}0.1)^2+y^2)^{0.5}$+π/2 rad, φ_2=-83.3$|y|$+π/2 rad.

The different initial phase will lead to different $h_{1/2}$ values of the phase modulator. The simulations of the array consider the air gap between neighbouring unit cells, in which the coupling effect may influence the results. Therefore, the change of the initial phase may change the coupling cases, which may improve the results, making the initial

phase optimization necessary.

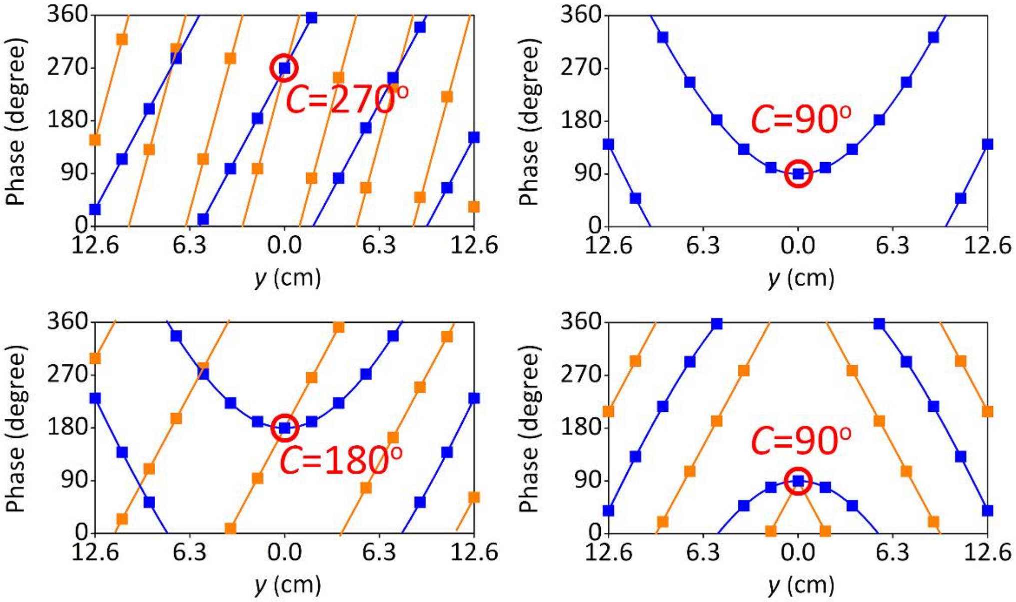

Supplementary Figure 7. The optimization of predesigned phase profile. Four pairs of phase profiles for Figs. 5-8 in the main text. The initial phases *C* are marked with the values of 270, 90, 180, 90 degrees, respectively.

Supplementary Note 7. Transverse resolution of the metascreen phase profile

In the original design, the unit cell width is *w*=1.8 cm, equal to *λ*/3 for the frequency of 6430 Hz. In order to have a higher transverse resolution, we show another design in Supplementary Figure 8 with a unit cell width of *w*=1 cm, equal to *λ*/5 for the working frequency of 6870 Hz. Supplementary Figure 8(a) shows the unit cell with two circulators. Supplementary Figure 8(b) exhibits the simulated phase responses with 720 rpm at 6870 Hz. Supplementary Figure 8(c) presents the values of $h_{1/2}$ for nonreciprocal extraordinary refractions, whose functionality is same as the design in Fig. 5 in the main text. Supplementary Figure 8(d) shows the schematic diagram of the array with 27 unit cells with the total width of 27 cm, same as original design. Supplementary Figures 8(e-f)

illustrate the nonreciprocal extraordinary refractions for forward direction (FD), and backward direction (BD), respectively. For FD, the plane wave incidence (0°) converts to extraordinary refraction (45°). For BD, the plane wave incidence (45°) converts to extraordinary refraction (-45°). The nonreciprocal effect for 27 unit cells is similar to the design of 15 unit cells, demonstrate that the 15 unit cells is sufficient to generate high-efficiency wavefront manipulations

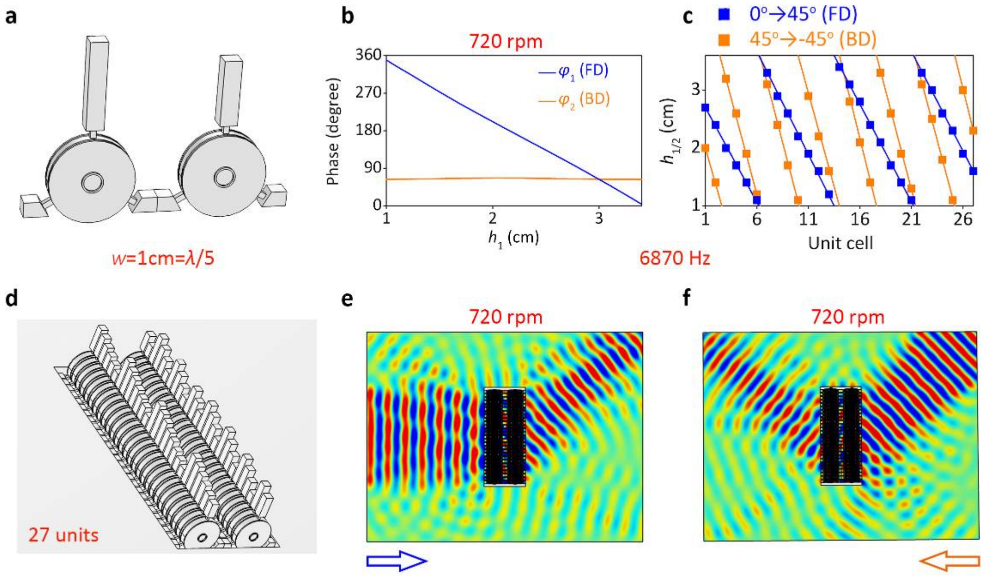

Supplementary Figure 8. Design with finer transverse resolution. (**a**) The unit cell design with unit cell width of w=1 cm, corresponding to $\lambda/5$ for the working frequency of 6870 Hz. (**b**) The simulated phase responses with 720 rpm at 6870 Hz. (**c**) The values of $h_{1/2}$ for nonreciprocal extraordinary refractions (**d**) The array with 27 unit cells (**e**) Nonreciprocal extraordinary refractions for FD (forward direction). (**f**) Nonreciprocal extraordinary refractions for BD (backward direction).

Supplementary Note 8. Detailed analysis of simulated and experimental results.

We also show more detailed analysis of simulated and experimental results for the two-faced manipulations by JAM in Figs. 7 and 8 in the main text, by mapping the quantitative curves for both simulation and experiment to show their function effects. For experimental results, the error bars representing the standard deviations for three sets of measured data are shown on the curves.

Supplementary Figure 9(a) shows the combination of acoustic focusing (FD) and extraordinary refraction (BD). For acoustic focusing (FD), we numerically and experimental map the acoustic pressure amplitude distributions along *y*-direction (*x*=0 cm, *y*=[-14 cm, 14 cm]). The simulated full width at half maximum is FWHM=3.8 cm (0.71λ). The experimental full width at half maximum is FWHM≈4.2 cm (0.79λ). For extraordinary refraction (BD), we numerically and experimental display the acoustic pressure amplitude directivity for (angle=[-90°, 90°]). The simulated normalized directional coefficients have a peak at -48°, and the measured normalized directional coefficients have a peak at 45°, agree with the theoretical value.

Supplementary Figure 9(b) shows the combination of acoustic diffusion (FD) and beam splitting (BD). For acoustic diffusion (FD), the simulated normalized directional coefficients are higher than 0.5 within the range of -63° to 63°, and the measured normalized directional coefficients are higher than 0.4 within the range of -80° to 80°, showing a good diffusion effect. For acoustic splitting (BD), the simulated and experimental normalized directional coefficients both have peaks near ±45°, showing good beam splitting effect.

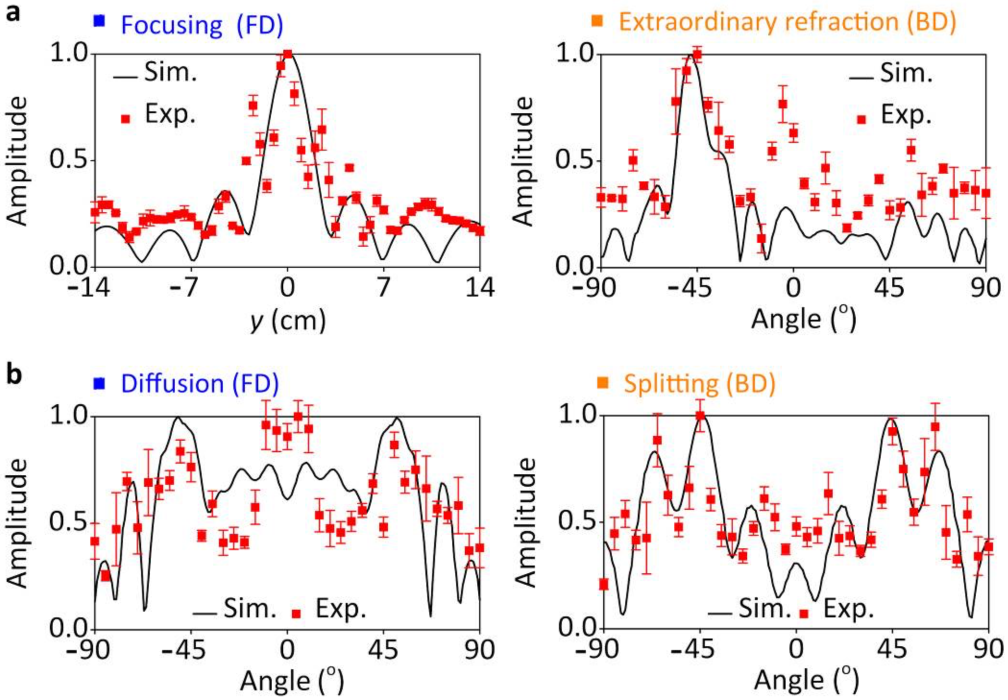

Supplementary Figure 9. Detailed analysis of simulated and experimental results. (**a**) Simulated and experimental acoustic pressure amplitude distributions along *y*-direction (*x*=0 cm, *y*=[-14, 14] cm) for acoustic focusing (forward direction, FD) and acoustic pressure amplitude normalized directional coefficients for (angle=[-90°, 90°]) extraordinary refraction (backward direction, BD). (**b**) Simulated and experimental acoustic pressure amplitude normalized directional coefficients for (angle=[-90°, 90°]) acoustic diffusion (BD) and acoustic beam splitting (FD). Error bars are shown in the figure, representing the standard deviations for three sets of measured data.
